# A Simple Exercise to Strengthen the Lower Esophageal Sphincter and Eliminate Gastroesophageal Reflux: An Autobiographical Case Report

**DOI:** 10.7759/cureus.24122

**Published:** 2022-04-13

**Authors:** Eric Karrfalt

**Affiliations:** 1 Research, Retired, McKean, USA

**Keywords:** esophageal resistance training, lower esophageal sphincter, autobiographical case report, gastroesophageal reflux, eliminate gastroesophageal reflux

## Abstract

A novel exercise is described for resistance training of the lower esophageal sphincter. Resistance is provided by gravity as food is swallowed and pushed up an incline into the stomach. The incline is established by kneeling with the head bowed lower than the stomach. After several months of daily repetitions, symptoms of gastroesophageal reflux ceased and the exercise was discontinued without relapse.

## Introduction

Gastroesophageal reflux results from weakness or relaxation of the lower esophageal sphincter (LES) [1]. Personal experience with this problem lead me to think about it, repeatedly. I came to entertain the hope that strengthening the LES might alleviate my reflux problem. Voluntary muscle can be strengthened by resistance training, but involuntary muscle like the LES characteristically cannot be strengthened in this manner. The esophagus, however, offers a special case. The swallowing process begins as a voluntary act which ultimately initiates a peristaltic wave of involuntary contractions through the smooth muscle in the lower two-thirds of the esophagus [2]. It occurred to me that that the LES might be strengthened if it were made to do a little extra work. The novel resistance training described here accomplishes this by requiring the LES to push food upward against gravity.

## Case presentation

I had been experiencing gastroesophageal reflux for a number of years. The symptoms became dramatically worse immediately after an endoscopy in 2016 in which an inexplicably large biopsy (estimated to be 0.3 x 0.5 x 0.2 cm, but not actually measured in three dimensions) was taken from my esophagus to rule out Barrett’s Esophagus. Nonetheless, all of my symptoms were eventually substantially controlled with ranitidine (150 mg three times a day) and a bed wedge. The ranitidine was a minor nuisance, but even after several refinements, the bed wedge remained intolerable.

Eventually, I devised the following regimen with the intent of providing the LES with some resistance training. The resistance was provided by positioning my head below my stomach in a kneeling posture. This required food being swallowed to be pushed up an incline. I began eating part of each breakfast (oatmeal) and sometimes lunch (a sandwich) in the exercise position. I would kneel on a platform (which happened to be 6 ½” high), take a normal mouthful, chew it as needed, and prepare to swallow. I would then lay my forearms and the backs of my hands on the floor, rest my head on my hands, and complete the swallowing process. With a little practice, I was soon able to initiate and complete the swallowing process with my head resting on my hands on the floor. I did not attempt to determine what the optimal height of the platform might be or if, indeed, any was necessary. 

Sixty-eight days after beginning daily LES exercises, I noticed that I could bend over at the hip and pull weeds in my garden without acid running into the back of my throat. This was not possible the previous year. I then tried sleeping without the bed wedge but found that it was still needed. I interpreted these observations to indicate that the LES was getting stronger, but still not strong enough. For about five more months I remained ambivalent as to whether the exercise would fully correct my problem and eventually sought help at the Cleveland Clinic. A 24-hour pH and manometry test was done, which yielded completely normal results. I then discontinued the use of the bed wedge and now have no symptoms that I can attribute to gastroesophageal reflux. I considered the possibility that a continuing training regimen might be necessary to maintain full LES function, so rather than risk a relapse, I continued to do the exercise a few times each week for a few months and then less frequently. I have not done the exercise at all for the past two years with no relapse.

My elimination of gastroesophageal reflux includes benefits beyond the ability to sleep comfortably on a horizontal surface. I can again do vehicle maintenance lying on my back with no esophageal discomfort. On one occasion I needed to do a minor repair to the gutter on my house in a location where the placement of a ladder was somewhat inconvenient. I was able to do the repair in about 10 minutes while lying prone looking downward near the edge of the sloping roof with no esophageal discomfort. Estimating from the pitch of the roof and my orientation with respect to the edge of the roof, my stomach was about two to three inches higher than my throat during this repair. These observations further attest to the restored competence of my LES.

## Discussion

In retrospect, I probably should have tested sleeping without the bed wedge at intervals after I first noticed an improvement in LES function. But once I had determined that I no longer needed the bed wedge, I would probably never have gotten the pH and manometry test, and this report, were it to be made, would have been based entirely on my interpretation of symptoms.

These observations clearly constitute proof of the concept of the LES exercise. Hopefully, others will benefit from this exercise, and their experiences may become the basis of a standardized protocol for its use. Many details, which I simply guessed at, could be optimized by systematic study. The height of or need for a kneeling platform, the frequency and duration that constitute effective training sessions, and a time frame in which results may be expected might all be determined. The texture and amount of food being swallowed may have some significance. It also remains to be seen if any contraindications exist for the LES exercise. This exercise is probably quite safe for any otherwise healthy person, but anyone beginning this exercise should use reasonable caution while developing his or her technique so as to avoid any discomfort.

## Conclusions

The resistance training exercise to strengthen the LES has many desirable attributes. It may eliminate the cause of gastroesophageal reflux rather than treat its symptoms and may well be a permanent solution to the problem. The exercise involves little or no risk or cost, and its use may be beneficial to many people.
